# The vertebral body growth plate in scoliosis: a primary disturbance of growth?

**DOI:** 10.1186/1748-7161-3-3

**Published:** 2008-01-26

**Authors:** Gregory Day, Kieran Frawley, Gael Phillips, I Bruce McPhee, Robert Labrom, Geoffrey Askin, Peter Mueller

**Affiliations:** 1 University of Queensland, Brisbane, Australia; 2 Royal Children's Hospital, Queensland, Australia; 3 Queensland Health Pathology Service, Herston, Australia; 4 Mater Children's Hospital, Brisbane, Australia

## Abstract

**Study Design and Aims:**

This was an observational pilot study of the vertebral body growth plates in scoliosis involving high-resolution coronal plane magnetic resonance (MR) imaging and histological examination. One aim of this study was to determine whether vertebral body growth plates in scoliosis demonstrated abnormalities on MR imaging. A second aim was to determine if a relationship existed between MR and histological abnormalities in these vertebral body growth plates.

**Methods:**

MR imaging sequences of 18 patients demonstrated the vertebral body growth plates well enough to detect gross abnormalities/deficient areas/zones. Histological examination of ten vertebral body growth plates removed during routine scoliosis surgery was performed. Observational histological comparison with MR images was possible in four cases.

**Results:**

Four of the 18 MR images demonstrated spines with normal curvature and normal vertebral body growth plates. In 13 scoliotic spines, convex and concave side growth plate deficiencies were observed most frequently at or near the apex of the curve. One MR image demonstrated a 55° kyphosis and no convex or concave side deficiencies. The degree of vertebral body wedging was independent of the presence of vertebral body growth plate deficiency. Histological abnormalities of the vertebral body growth plates were demonstrated in four with MR imaging abnormalities.

**Conclusion:**

This study demonstrated MR image abnormalities of scoliotic vertebral body growth plates compared to controls. A qualitative relationship was demonstrated between MR imaging and histological abnormalities. The finding that vertebral body growth plate deficiencies occurred both on the convex and concave sides of the spine, closest to the apical vertebra of the scoliosis curve, implied that they are less likely to be the result of adaptive changes to the physical forces involved in the scoliotic deformity. One explanation is that they represent a primary disturbance of growth.

## Background

**Figure 1:**
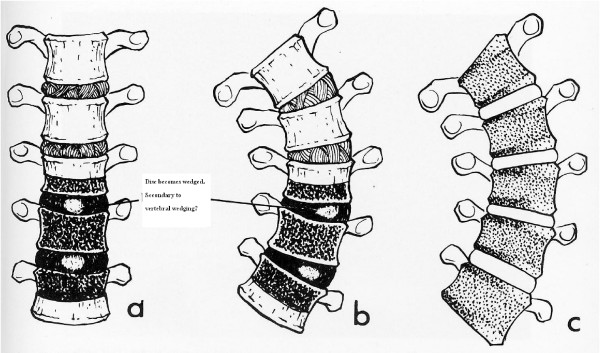
Hueter-Volkmann Principle of Differential Growth.

Structural scoliosis is defined as a lateral curvature of the spine, involving a spinal rotation towards the concavity and is classified according to cause – congenital, neurological, neuro-muscular, post-traumatic and idiopathic. The etiology of all but idiopathic is self-evident and the progression of deformity is popularly believed to be linked to the mechanical modulation of growth theory [1,2]. It is based on the Hueter-Volkmann principle of differential growth through differential pressure loading on the growth plate [3] (Figure 1). Eular's Law of viscoelastic buckling of a spine in the coronal and transverse planes leading to a lateral bend and axial rotation/torsional buckling respectively, is a mechanical explanation of the forces acting on the vertebral body growth plates as well as the entire spinal column [4,5]. Logically, the progression of the deformity in idiopathic scoliosis should be governed by similar principles. The trigger causing the evolution of the deformity remains a mystery.

There is broad agreement that vertebral wedging in the frontal plane is present in all types of scoliosis and that wedging is maximal at the apex of the spinal curve [6-10]. In idiopathic thoracic scoliosis, the adjacent intervertebral discs are wedged to a lesser degree than the vertebrae, implying that disc wedging occurred secondarily [11]. In idiopathic scoliosis, the presence of intra-vertebral rotation and disproportionate anterior spinal overgrowth suggest that asymmetrical growth has occurred [12-17]. All types of scoliosis progress faster following the pubescent growth spurt, indicating that the shape of the vertebral bodies changes most rapidly with vertebral growth [18,19]. 'Because scoliosis progresses during the pubescent growth spurt, it is likely that the vertebral body growth plate is a major factor in the development of the scoliosis deformity' [20]. From childhood, vertebrae grow through thin growth plates on the superior and inferior vertebral end-plates and from neuro-central, articular process and spinous process synchondroses [21-23]. Magnetic resonance (MR) characteristics of normal neuro-central synchondroses and skeletally mature vertebral end plates have been recently reported [24-26]. MR characteristics of normal and scoliotic vertebral body growth plates have not been reported and one aim of this study was to determine whether vertebral body growth plates in scoliosis demonstrated abnormalities on high-resolution coronal plane MR imaging. Simultaneously, is was possible to determine whether the vertebral body growth plate abnormalities could be linked to wedging deformity of individual vertebral bodies.

Observational studies of the histology of vertebral body growth plates in idiopathic scoliosis have reported abnormalities [27-29], which were thought to represent "premature partial closure of the growth plate" [30]. A second aim of this study was to confirm these previous observations and to determine if a relationship existed between MR and histological abnormalities in vertebral body growth plates in idiopathic and other scolioses.

## Methods

### Magnetic Resonance Imaging

**Figure 2:**
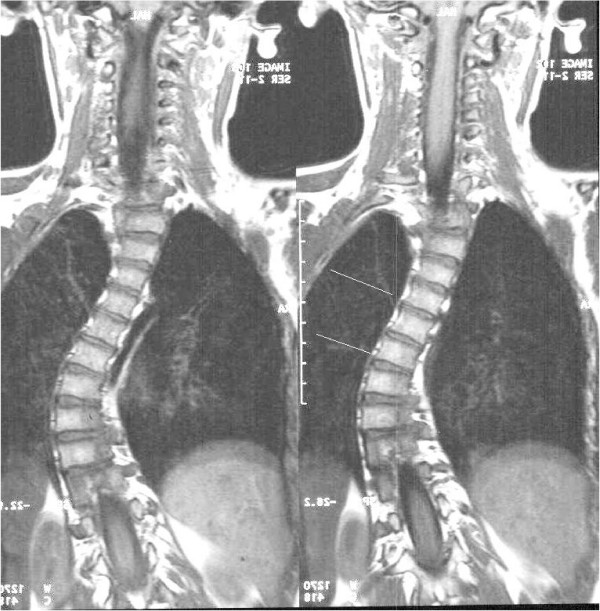
MRI T1-weighted Sequence, Coronal Image. Idiopathic Scoliosis Patient #3. Superior line indicates central zone deficiency and inferior line indicates convex zone growth plate deficiency.

**Figure 3:**
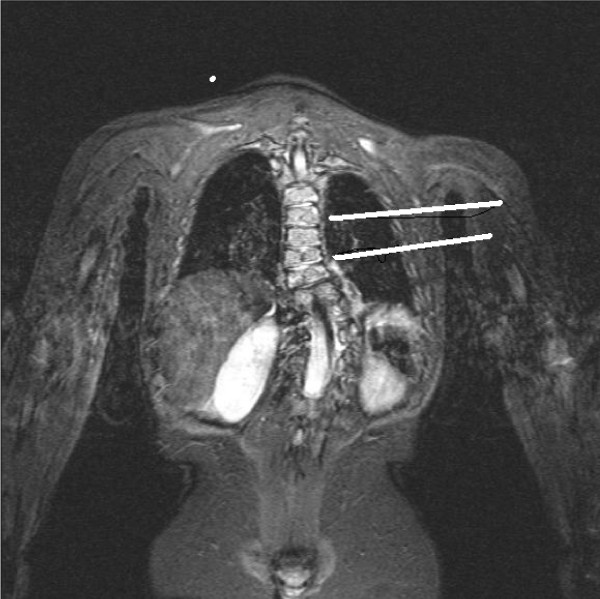
MRI T2-weighted Sequence, Coronal Plane Fat Saturation image. Complex Congenital Scoliosis Patient # 5. Superior line demonstrates concave zone growth plate deficiency and inferior line demonstrates a straight growth plate.

**Figure 4:**
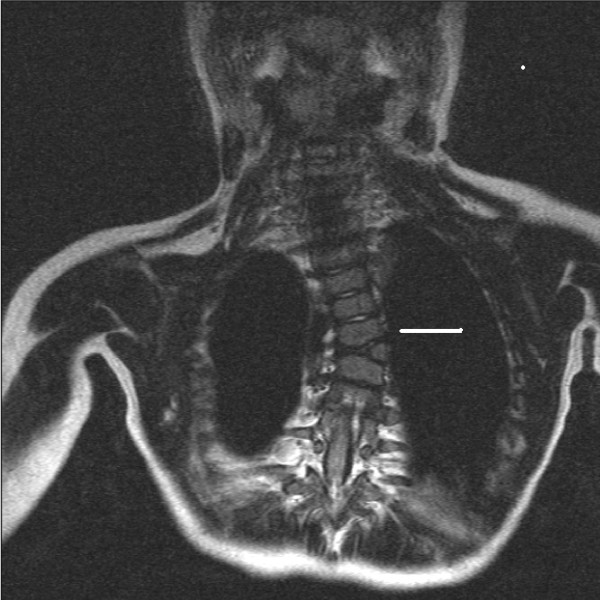
MRI T2-weighted Sequence, Coronal Plane Image. Congenital Scoliosis from Hemivertebra Patient # 21. MRI demonstrates convex zone deficiency in the vertebral body growth plate.

High-resolution coronal plane spinal MR imaging of 29 patients was studied to determine whether the vertebral body growth plates were clearly visible and if zones of deficiency of height were visually detectable. A 1.5 Teslar Siemens system (Erlanger, Germany) with a 4.1AGFA IMPAX software package was used. This allowed digital measurement of angles. Eighteen of the 29 series of images demonstrated vertebral body growth plates clearly and patient demographics were tabulated (Table 1). Of the 18, 13 had scoliosis and four had a straight spine, acting as a control. Another patient demonstrated a 55° kyphosis and no detectable vertebral body growth plate abnormality on coronal plane imaging. Six patients had idiopathic and seven had scoliosis from other causes. The mean patient age was 10 (Range 1.5–16.3 years). There were eleven female and seven male patients. Data from each set of imaging was collected and analysed. Documented variables included the Cobb angle, the presence and degree of segmental vertebral and disc wedging and vertebral growth plate zone deficiencies. Growth plate deficiency was defined as a visible gross reduction in the thickness of a zone (Figures 2, 3, 4). The vertebral body growth plate was divided into three zones – concave and convex sides of the curve and the central portion.

**Table 1:** 

Patient number	Syndrome	Age at MRI(years)	Site curve	Cobb angle
1	Idiopathic Scoliosis	13	T5-T12 R	64
2	Osteogenesis Imperfecta	16.3	T7-L2 R	58
3	Idiopathic Scoliosis	12.7	T6-T11 R	52
			T11-L4 L	58
5	Congenital scoliosis	1.5	T10-L2 L	72
6	Cervico-thoracic hydromyelia	9.1	T6-L1 R	40
			L1-L5 L	38
7	Neurological condition	4.8	Straight	control
8	Neurofibromatosis	10	Straight	control
10	VATER	14	T5-T10 L	55
			T10-L3 R	52
18	Idiopathic Scoliosis	11.8	T5-L1 R	43
19	Sacral agenesis	7.5	min. curve	control
21	T 7 hemivertebra	2.4	T5-T10	35
22	Fractured C7	13.3	Crush # C7	control
23	Neurofibromatosis Type 1	11.4	Kyphosis 55	control
25	Prader Willi syndrome	11.5	T5-11 R	75
26	Idiopathic Scoliosis	12.8	T4-10 R	50
27	Idiopathic Scoliosis	5.9	T6-11 R	43
28	Turner syndrome	11	T3-10 R	30
29	Idiopathic Scoliosis	12.1	T4-10 R	16

### Histopathological Examination

**Figure 5:**
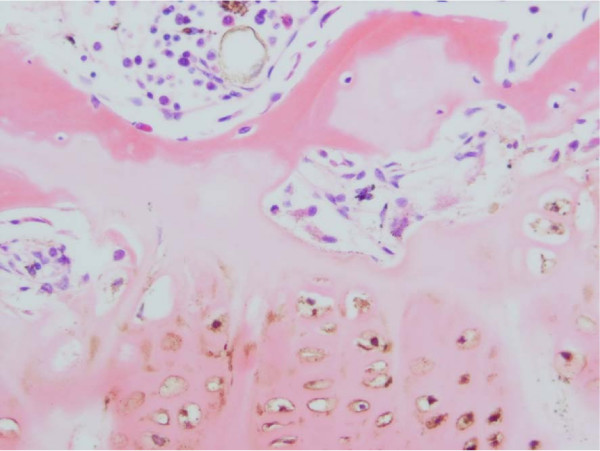
Haematoxylin & Eosin Stained Section Mag. ×200 from the Marked Straight Vertebral Body Growth Plate from the MR Image of Congenital Scoliosis Demonstrated in Figure 3. Minimally disordered vertebral body growth plate with almost normal columns of chondrocytes.

**Figure 6:**
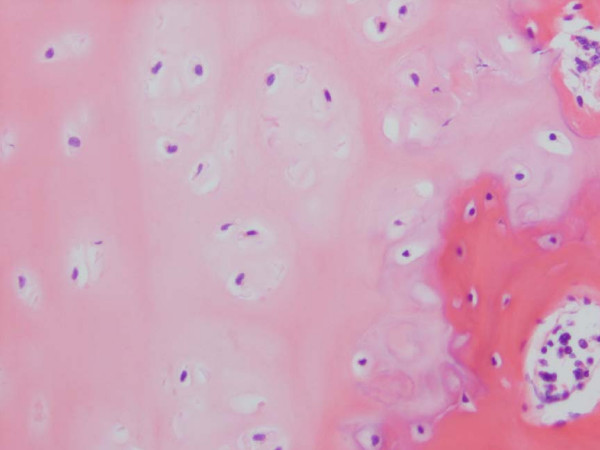
Haematoxylin & Eosin Stained Section of Congenital Scoliosis Vertebral Growth. Plate of MR Image in Figure 4 (convex zone deficiency). Slightly reduced activity and mildly disorganised columns of chondrocytes.

**Figure 7:**
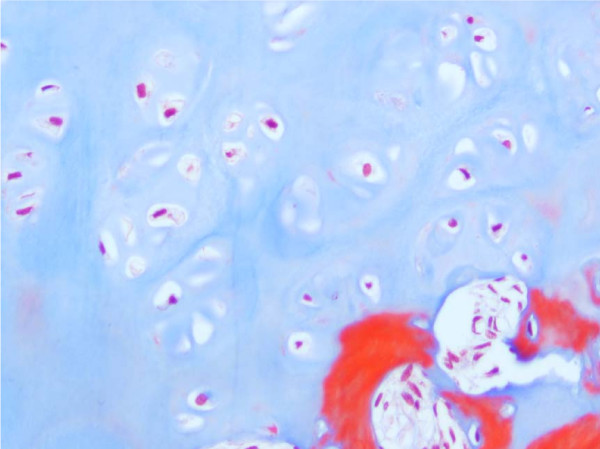
Masson Trichrome Stained Section of the Same Vertebral Growth Plate as in Figures 4 and 6. Slightly reduced activity and mildly disorganised columns of chondrocytes.

**Figure 8:**
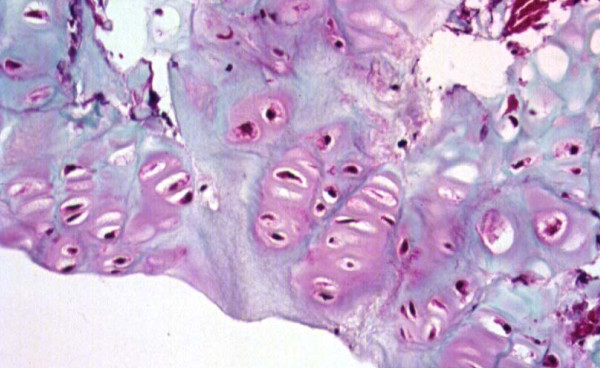
Masson Trichrome Stained Section Mag. ×200 From the Convex Zone Growth Plate. Abnormality of Idiopathic Scoliosis Patient #3. The columns of chondrocytes are mildly disordered.

**Figure 9:**
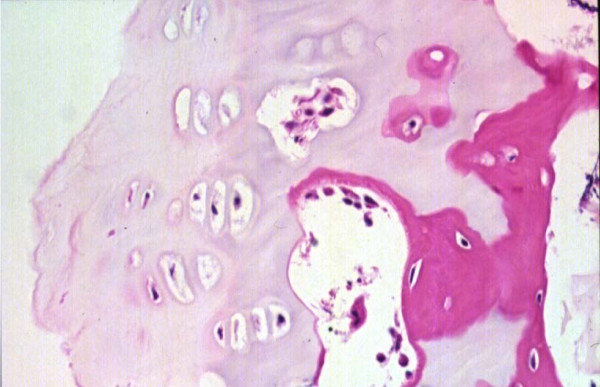
Haematoxylin & Eosin Stained Section Mag. ×200 from Idiopathic Scoliosis Patient # 26. There is reduced activity of the concave aspect of the vertebral body growth plate with shorter columns of chondrocytes.

**Figure 10:**
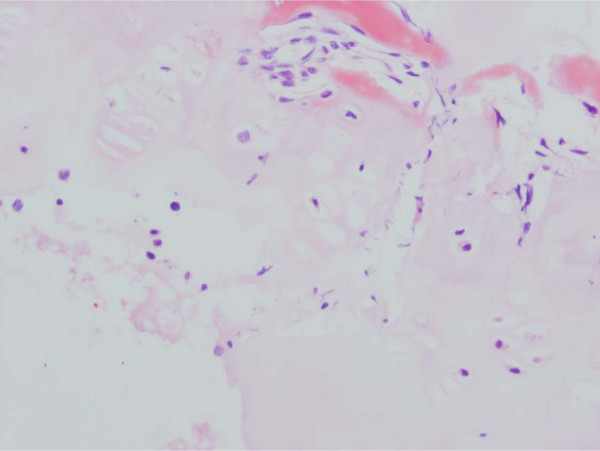
Haematoxylin and Eosin Stained Section Mag. ×100. Mildly disordered columns of chondrocytes in the vertebral body growth plate in neurofibromatosis patient # 11.

**Figure 11:**
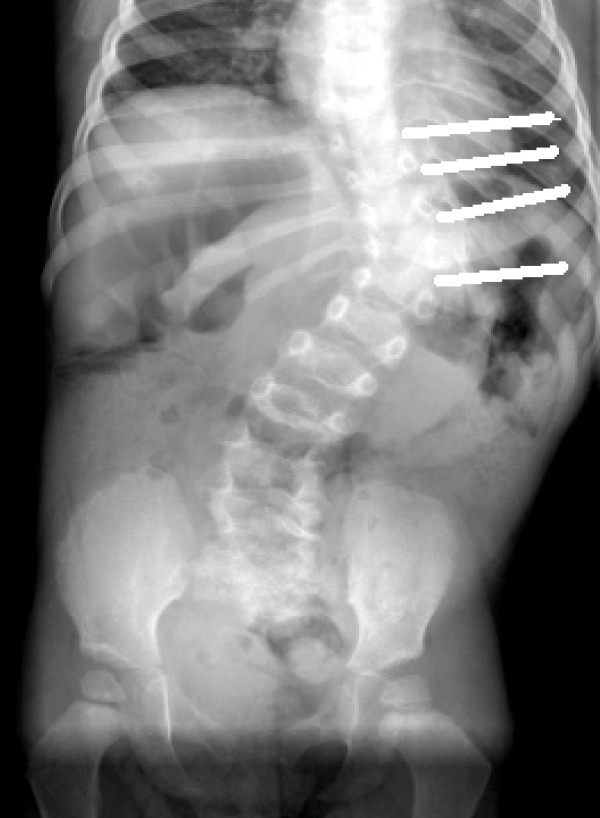
PA Cong. Scoliosis Patient #5 with Removed Intervertebral Discs Marked.

Paraffin blocks were constructed from ten vertebral body growth plates removed during routine scoliosis surgery. These growth plate specimens included five congenital scoliosis (including patients # 5, 21), four idiopathic (including patients # 1,3,18,26) and one non-dystrophic neurofibromatosis scoliosis (patient #8) (Figures 5, 6, 7, 8, 9, 10). The four idiopathic scoliosis patients were above the 50^th ^percentile height for their age. Pre-operative AP and lateral radiographs of the spine were marked to demonstrate the disc and growth plates removed during surgery (Figure 11). The entire intervertebral disc and growth plate was removed in each case and sections were obtained from the convex and concave sides. Generally, the convex annulus fibrosis, ring apophysis and vertebral growth plates were removed in one block, whilst the concave-side growth plates consisted of curettings. Paraffin blocks with the larger intact convex-side disc/vertebral growth plate were identified prior to sectioning. Histological staining by Haematoxylin & Eosin and Masson Trichrome was performed on paraffin sections 4–5 μm thick. Histopathology reports were issued with the pathologist (GP) blinded to the clinical diagnosis.

The findings on MR imaging and histopathology were illustrated for two patients with idiopathic scoliosis and two with congenital scoliosis.

## Results

### Radiologic (MR) Examination

**Figure 12:**
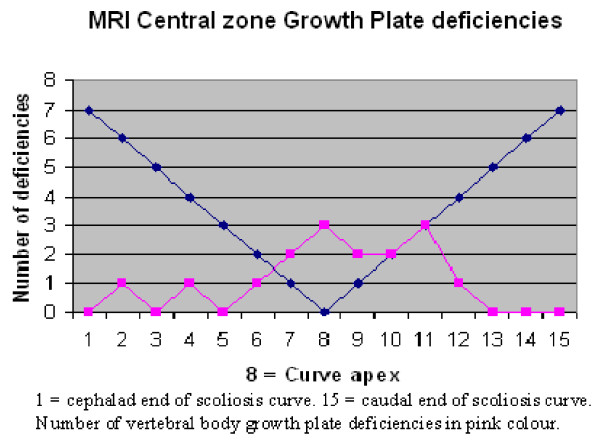


**Figure 13:**
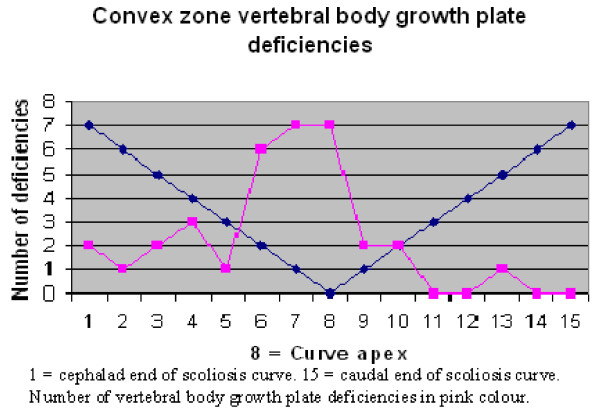


**Figure 14:**
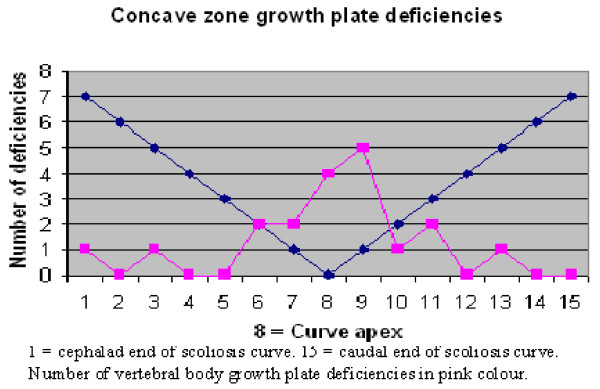


T1-weighted sequences were the best images to visualize the vertebral body growth plates on high-resolution coronal plane spinal MRI. Fourteen of the MRIs were T1 and four were T2-weighted sequences. No vertebral body growth plate deficiencies were demonstrated in the five control spines on coronal plane imaging. A total of 19 concave, 16 central and 34 convex zone growth plate deficiencies were noted in 13 scoliotic spines. The distribution of vertebral growth plate deficiencies in relation to the apical vertebra of the scoliotic curves is depicted in Figures 12, 13, 14. Most of the deficiencies occurred at or near the apex of the scoliosis curve and followed a trend towards a normal Bell curve distribution. Because of relatively small numbers and a slightly skewed distribution from the apex, statistical analysis of the distribution of these growth plate deficiencies was believed to be unhelpful.

**Figure 15:**
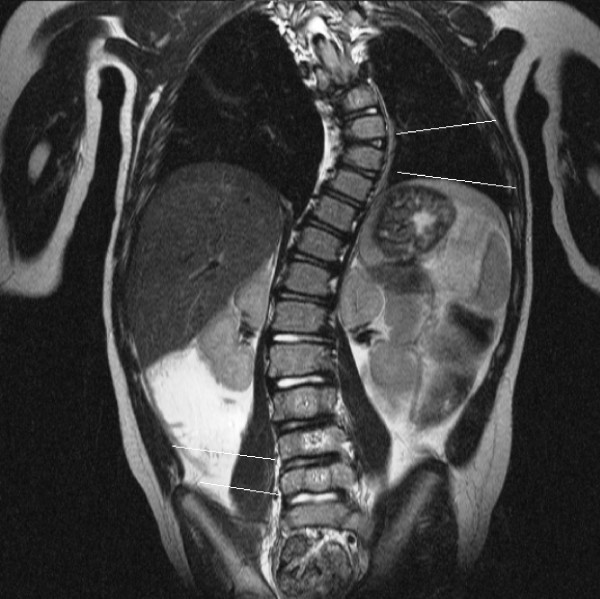
MRI T2-weighted Coronal Plane Image Idiopathic Scoliosis Patient #26. Schmorl's nodes on superior and inferior marker lines compared to normal discs at the adjacent levels.

In scoliotic spines, accurate measurement of vertebral body wedging was hampered by the presence of convex-side Schmorl's nodes in five patients (Figure 15). Curiously, the presence of the Schmorl's nodes accounted for the presence of most of the convex zone vertebral body growth plate deficiencies. Measurements were recorded and the combined wedging of all vertebrae and intervertebral discs in the scoliotic curve of each individual spine was found to be similar (not statistically different).

### Histopathologic Examination

Specimens from congenital scoliosis demonstrated mildly disordered columns of chondrocytes and macroscopic reduction of the volume of the growth plate, corresponding to the vertebral body growth plate abnormalities demonstrated on MR imaging in two spines. Reasonably normal columns of chondrocytes were demonstrated when the MR image demonstrated straight vertebral body growth plates (Figures 5, 6, 7).

One specimen from a patient with idiopathic scoliosis demonstrated a mild abnormality of columns of chondrocytes on the convex side of the vertebral body growth plate (Figure 8). Three specimens from patients with idiopathic scoliosis demonstrated reduced activity on the concave side of the growth plate (Figure 9). Normal columns of chondrocytes were demonstrated in other zones of the growth plate. The specimen from a patient with neurofibromatosis demonstrated irregular and shortened columns of chondrocytes on the concave side of the growth plate (Figure 10).

## Discussion

### MR Imaging of Vertebral Growth Plates

Normative MR imaging data of the thickness and quality of vertebral body growth plates in straight spines and scoliosis has not been reported. The new 1.5 Teslar system at the authors' institution lacks ultra-fine resolution, but has the capacity to demonstrate reduction in the height of zones within each vertebral body growth plate. The authors believed that ultra-fine resolution was not an absolute necessity for this observational study.

The observation of Schmorl's nodes in idiopathic scoliosis on MR imaging was only recently described and their pathogenesis was not discussed [31]. In this study, the presence of Schmorl's nodes on the convex sides of the vertebrae was curious, as this side is subject to less force from gravity than the concave side. Future studies with a larger cohort may help to determine the pathogenesis of Schmorl's nodes in idiopathic scoliosis.

In this study, there was no relationship between the presence of convex growth plate deficiencies and the degree of wedging of the 34 involved vertebrae. It is peculiar that vertebral body growth plate deficiencies were observed on the convex side of the vertebrae in scoliosis, as this implied reduced growth on the convex side. Just over 2/3 of the concave zone growth plate deficiencies occurred near the apex of the scoliotic curves. This may imply either a premature partial growth plate fusion or reduced growth plate activity conforming to the Hueter-Volkmann Law, regarded as a secondary adaptive change [3]. However, the fact that central, as well as concave and convex zone vertebral body growth plate deficiencies were observed to be more frequent near the apex of the curve implied that they are probably not adaptive changes secondary to differential pressure loading on areas of the vertebral body growth plate [29,32].

In this study, no difference between the combined vertebral body and intervertebral disc wedging was demonstrated in the curves of individual patients with scoliosis, which contrasts with the findings of previous studies [11,33]. In the former study, adjacent intervertebral discs were wedged to a lesser degree than vertebrae in idiopathic thoracic scoliosis, implying that disc wedging occurred secondarily [11]. The latter compared the growth of T8 and L4 vertebrae in ambulant children vs non-ambulant children with cerebral palsy, and concluded that intervertebral disc wedging was the primary reason for the development of scoliosis in cerebral palsy [33]. The previous studies were based on plain radiographs and were longitudinal in nature. More longitudinal studies with MR imaging on a larger cohort of children with scoliosis may help to resolve the issue regarding whether vertebral body or intervertebral disc wedging develops first.

### Histopathology of Vertebral Body Growth Plates

The reduced activity of columns of chondrocytes on the concave side of the idiopathic scoliotic vertebrae was consistent with previous findings in a larger cohort and was in accordance with a belief that a premature partial fusion of the vertebral body growth plate had occurred [30]. However, premature growth plate closure would normally lead to shortened stature, which is the opposite of the normally observed tall stature in females with idiopathic scoliosis. Both idiopathic scoliosis patients with illustrated histopathology of the growth plates in this study were above the 50^th ^percentile height for their age.

The disordered columns of chondrocytes in the vertebral body growth plates of congenital scoliosis and the non-dystrophic scoliosis associated with neurofibromatosis were to be expected, as each condition is associated with known disorders of growth. In this study, disordered columns of chondrocytes were also observed in one idiopathic scoliosis patient. The growth plate appeared to be normal in height and activity. This has not been previously reported and future research on a larger cohort may help to define a relationship between individual vertebral body growth and histopathological abnormalities of the growth plates.

### Comparison of MR Imaging and Histopathology of the Vertebral Body Growth Plates

Although 10 histological specimens were studied, comparison with only four MR images was illustrated because some of the original 29 MR imaging studies were either incomplete or had resolution which did not allow scientific scrutiny. In this study, reduced activity of the chondrocytes on the concave zone of the vertebral body growth plates in two with idiopathic scoliosis corresponded to the MR imaging findings of a reduction in vertebral body growth plate height. These findings were similar to previous reports [27,28,30]. Findings of disorganisation within the vertebral body growth plates in congenital scoliosis were also consistent with the MR imaging observations in two patients. Observation of the MR images of the vertebral body growth plates could not locate the deficiencies/abnormalities precisely in three planes. For this reason, the observed growth plate changes were classified as deficiencies of one zone. These observations are open to interpretation.

### Interpretation of the Observations from this MR Imaging/Histopathology Study

If the observed *deficiencies *of vertebral body growth plates were actually normal zones of growth plates, then the remainder of the vertebral body growth plates could be increased in height (compared to normal size), implying overactivity. This would have obvious implications for the hypothesis of disproportionate vertebral body growth and lengthening of the anterior column observed in idiopathic scoliosis. When magnetic resonance systems capable of providing ultra-high resolution coronal plane spinal imaging are commonly available, data may become available which may help to better explain the evolution of the idiopathic scoliosis deformity.

## Authors' contributions

GD designed the study, IBM contributed to the study design and contributed growth plates, PM (radiologist) designed the coronal plane imaging sequences and KF (radiologist) interpreted the observations. GP (pathologist) interpreted the histopathology and photographed all specimens. RL and GA contributed specimens and assisted in quality control for specimen orientation. All authors read and approved the final manuscript.
